# Effects of Preexisting Psychotropic Medication Use on a Cohort of Patients with Ischemic Stroke Outcome

**DOI:** 10.1155/2020/9070486

**Published:** 2020-09-22

**Authors:** Adalia H. Jun-O'Connell, Dilip K. Jayaraman, Nils Henninger, Brian Silver, Majaz Moonis, Anthony J. Rothschild

**Affiliations:** ^1^Departments of Neurology, University of Massachusetts Medical School, Worcester, MA, USA; ^2^Neurology Department, Tower Health Medical Group, University of Massachusetts Medical School, Worcester, MA, USA; ^3^Department of Psychiatry, University of Massachusetts Medical School, Worcester, MA, USA

## Abstract

**Background:**

Several studies investigated the use of selective serotonin reuptake inhibitors (SSRI) after ischemic stroke to improve motor recovery. However, little is known about the effects of preexisting psychotropic medication use (PPMU), such as antidepressants, on a long-term ischemic stroke functional disability.

**Objective:**

We sought to determine the prevalence of PPMU and whether PPMU relates to the long-term clinical outcome in a cohort of patients presenting with acute ischemic strokes.

**Methods:**

We retrospectively analyzed 323 consecutive patients who presented with an acute ischemic stroke in a single institution between January 2015 and December 2017. Baseline characteristics, functional disability as measured by the modified Rankin Scale (mRS), and major adverse cardiovascular complications (MACE) within 365 days were recorded. The comparison groups included a control group of ischemic stroke patients who were not on psychotropic medications before and after the index ischemic stroke and a second group of poststroke psychotropic medication use (PoMU), which consisted of patients started on psychotropic medication during the index admission.

**Results:**

The prevalence of PPMU in the studied cohort was 21.4% (69/323). There was a greater proportion of females in the PPMU than in the comparison groups (*P* < 0.001), while vascular risk factors were similar in all groups, except for an increased presence of posterior circulation infarcts in the PPMU (37.4% vs. 18.8%, *P* < 0.001). Among the patients with available 1-year follow-up data (*n* = 246), we noted significantly greater improvement in stroke deficits, measured by National Institute of Health Stroke Scale (NIHSS) between PPMU and PoMU vs. control (3 (0-7) versus 1 (0-4), *P* = 0.041). The 1-year mRS was worse in PPMU and PoMU compared to the control group (2 (IQ 1-3) vs. 2 (IQ 0-3) vs. 1 (IQ 0-2), respectively, *P* = 0.013), but delta mRS reflecting the degree of mRS improvement showed no significant difference between any PMU and control patients (*P* = 0.76). There was no statistically significant difference in MACE.

**Conclusion:**

PPMU in ischemic stroke is common; it can be beneficial in ischemic stroke in the long-term clinical outcome and is not associated with increased risks of MACE.

## 1. Introduction

Stroke is the third leading cause of worldwide disability [1] and a leading cause of serious long-term disability in the US, reducing mobility in more than half of its survivors aged 65 and older [2]. Motor deficits affect up to 82% of patients after stroke and are associated with decreased quality of life [3]. Several neurotransmitters, specifically norepinephrine [4] [5] [6], dopamine [7] [8], and serotonin [9] [10, 11] have been implicated in the modulation of motor recovery after brain injury. A meta-analysis of combined animal and human researches suggests that antidepressants, such as selective serotonin reuptake inhibitors (SSRI), play a crucial role in poststroke neurological recovery through its probable action on a regenerative process [12]. Subsequently, there has been an increased interest in the role of psychotropic drugs to potentially improve poststroke motor recovery [3].

Preexisting psychotropic medication use (PPMU) prior to the onset of an ischemic stroke on long-term stroke outcome is unknown. SSRI have been studied in several randomized control studies, yielding conflicting results regarding the effect of the medication on outcomes after an ischemic stroke [11, 13–15]. Furthermore, the effects of PPMU on acute stroke severity and poststroke outcome have not been well studied. To our knowledge, there has been only one cohort study that investigated the association between SSRI use prior to an ischemic stroke and subsequent outcome within 30 days [16].

In the current study, we sought to determine the prevalence of PPMU in a cohort of patients presenting with an acute ischemic stroke and whether PPMU was associated with the initial stroke severity as assessed by the admission National Institute of Health Stroke Scale (NIHSS) score. Secondary objectives were to determine the association between PPMU with long-term functional disability as assessed on the modified Rankin Scale (mRS), as well as major adverse cardiovascular events (MACE).

## 2. Methods

### 2.1. Study Cohort

The study was approved by the local Institutional Review Board, and a Health Insurance Portability and Accountability Act waiver of informed consent was approved. We retrospectively analyzed prospectively accrued adult patients (greater than age 18 years) who were evaluated at the University of Massachusetts Medical Center between January 2015 and December 2017. We followed the Strengthening the Reporting of Observational Studies in Epidemiology guidelines (http://www.strobe-statement.org) [17].

### 2.2. Definitions

We defined ischemic stroke as an episode of neurological dysfunction due to a focal CNS infarction, attributable to ischemia [18].

Psychotropic medications were defined as drugs used to treat psychiatric disorders [19]. The specific psychotropic medications examined in this study included drugs that target dopaminergic, norepinephrinergic, and serotonergic neurotransmitters, including antidepressants (tricyclic antidepressants, SSRIs, serotonin-norepinephrine reuptake inhibitors, serotonin antagonist and reuptake inhibitors, monoamine oxidase inhibitors), amphetamines, lithium, and select atypical antipsychotics (quetiapine, aripiprazole, and ziprasidone for examples, which may enhance serotonergic transmission) [19].

**Figure 1:**
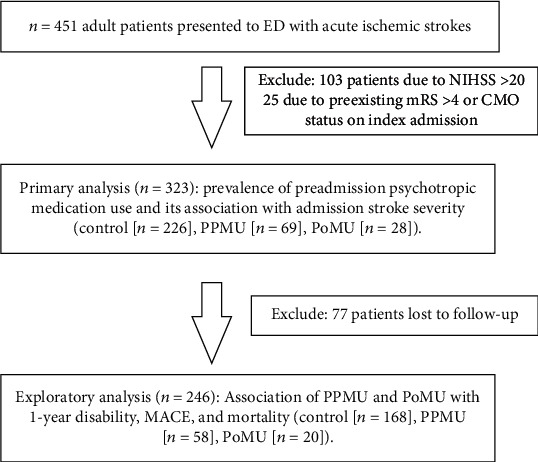
Patient flow chart.

Based on the use of psychotropic medication before and after the index stroke, we stratified patients to three groups (Figure 1): first, preexisting psychotropic medication use (PPMU), which included patients who were treated with a psychotropic medication prior to the onset of the acute ischemic stroke and were continued on them after the index stroke; second, poststroke psychotropic medication use (PoMU), which included patients who were started on psychotropic medication during the index admission; and third, control, which included patients who were neither treated with psychotropic medications before nor after the index stroke. To minimize potential expectation bias, a subclassification of study groups was carried out blind to any follow-up data.

A favorable 1-year functional outcome was defined as a modified Rankin Scale (mRS) score ≤ 2 [20]. We defined the degree of functional deficit recovery as the difference between the admission NIHSS minus the 1-year NIHSS score (delta NIHSS), whereby larger numbers indicate greater deficit improvement. The mRS assessment in the poststroke period was assessed by a stroke-trained physician certified in mRS via in-person. When the mRS was not available, the mRS was reconstructed from the case description based upon the mRS criteria [21]. All diagnoses were first established by treating physicians and then reassessed by a trained vascular neurologist (D.J.) after independent chart reviews of the medical records. A double-boarded psychiatrist and neurologist (A.J.O.) adjudicated uncertain cases, and remaining discrepancies were resolved by consensus.

### 2.3. Exclusion

We excluded patients with severe stroke deficits (NIHSS > 20) with expected poor prognosis, substantial premorbid disability (mRS > 4), patients who died in house or were discharged to hospice. Patients lost to follow-up were excluded from our exploratory analyses.

### 2.4. Data Collection

Patient demographics, comorbidities, preadmission medications, admission NIHSS, admission mRS, and imaging data (MRI brain and/or CT head) were collected for all patients by neurology trained physicians [22]. All included patients (*n* = 323) underwent a brain CT. An additional imaging with brain MRI (*n* = 301, 93.2%) was done at the discretion of the treating physician at the time of the index admission.

### 2.5. Outcomes

The primary goals were to identify the prevalence of PPMU in acute ischemic stroke and its association with admission stroke severity as graded by NIHSS. For the purpose of this analysis, we compared PPMU with non-PPMU (controls + PoMU) patients.

In a subsequent exploratory analysis, we sought to determine whether psychotropic medication use was associated with 1-year disability (defined as mRS > 2), delta mRS (degree of mRS improvement from baseline to 1 year to account for the degree of prestroke mRS), delta NIHSS (degree of NIHSS score improvement from baseline to 1 year to account for potential confounding by indication as patients with worse acute NIHSS were more likely started on psychotropic medication), and major adverse cardiac events (MACE) and its individual components of recurrent nonfatal stroke, nonfatal myocardial infarction, and cardiovascular death. Given prior clinical trial data suggesting beneficial effect on motor recovery with PoMU [11], we conducted an additional exploratory analysis to determine whether any psychotropic medication use (any PMU, i.e., combined PPMU and PoMU) was associated with improved 1-year disability and neurological deficit recovery (as assessed by the delta NIHSS) at one year when compared to controls.

### 2.6. Statistical Analyses

Data are reported as median (interquartile range) unless otherwise stated. Univariate comparisons were performed with *χ*^2^, Fisher exact, Mann–Whitney *U* tests, and Kruskal Wallis ANOVA on ranks as appropriate. Two-sided significance tests were used throughout, and a two-sided *P* < 0.05 was considered statistically significant unless stated otherwise. To calculate corrected levels of significance in cases of multiple comparisons in the univariate analyses, adjusted significance level was calculated using the Bonferroni correction. All statistical analyses were performed using the IBM SPSS Statistics version 20.0.0 (IBM, Armonk, NY).

## 3. Results

### 3.1. Study Participants and Prevalence of Psychotropic Medication Use

Overall, 323 patients fulfilled the study criteria and were included in the data analysis (Figure 1). Of these, 69 (21.4%) patients were on PPMU, 28 (8.7%) patients were on PoMU, and 226 (69.9%) patients served controls. The details on the used psychotropic medications stratified by PPMU and PoMU are shown in Table 1.

### 3.2. Clinical Characteristics Associated with PPMU

The baseline characteristics of the studied patient population as stratified by PPMU versus non-PPMU are shown in Table 2. The prevalence of female gender in PPMU was higher than in other groups (68.1% vs. 37.4%, *P* < 0.001). Moreover, the PPMU group had similar prevalence of preexisting vascular risk factors compared to the non-PPMU group, including hypertension, diabetes mellitus, prior history of TIA and stroke, atrial fibrillation, coronary artery disease, and peripheral vascular disease (*P* > 0.05), and similar use of stroke prevention medications (*P* > 0.05).

### 3.3. Association of PPMU with Initial Stroke Severity

Overall, baseline characteristics of PPMU and non-PPMU groups were similar except for a higher preadmission mRS and more frequent posterior circulation stroke location in PPMU patients (*P* < 0.05, each, Table 2). With regard to the initial stroke severity, we found no significant difference in the admission NIHSS between PPMU and non-PPMU groups (median 3 (IQ 1-8.5) vs. 3 (IQ 1-7), *P* = 0.352). Similarly, there was no difference in the final infarct volume and length of hospital stay (*P* > 0.05, each).

### 3.4. Exploratory Analysis of 1-Year Outcome Events

A total of 246 (76.2%) patients had 1-year outcome data available for analysis. There was no significant difference in the proportion of patients lost to follow-up between groups (*P* > 0.05).  Table 3 depicts the secondary outcome events in the three defined groups.

We found that the clinical deficit severity as measured by the NIHSS at one year was significantly worse in PoMU compared to control and PPMU patients, respectively (median 1.5 vs. 0 and 1; *P* = 0.035). To account for potential confounding by indication (i.e., patients with worse acute NIHSS were more likely started on psychotropic medication), we also examined the degree of NIHSS improvement from admission to 1 year (delta NIHSS). In this analysis, there was a trend towards greater NIHSS improvement between PPMU and PoMU versus control, though this did not reach significance (Table 3). In a separate analysis of any PMU versus control, any PMU patients had an overall greater focal deficit recovery than controls as assessed by the delta NIHSS (3 (0-7) versus 1 (0-4), *P* = 0.041).

When we examined the degree of functional disability as measured by the mRS, we found that patients in both the PPMU and PoMU had significantly worse 1-year mRS than the control group 2 (1–3) vs. 2 (0-3) vs. 1 (0-2), respectively, *P* = 0.013). When we accounted for the degree of prestroke mRS by calculating the degree of mRS improvement from baseline to 1 year (delta mRS), this effect was attenuated when compared across all three groups (*P* = 0.046), and there was no significant difference between any PMU and control patients (*P* = 0.76).

Finally, we found no statistically significant difference between the 3 groups in the rates of 12-month MACE (recurrent nonfatal stroke, nonfatal myocardial infarction, and cardiovascular death) (*P* > 0.05, each, Table 3). The results were not meaningfully different when we compared controls with any PMU (not shown).

## 4. Discussion

Our study is important as it addresses the potential association between preadmission psychotropic medication use in ischemic stroke and long-term disability in a select cohort of patients presenting with ischemic strokes. This is relevant as psychotropic medication in stroke is becoming more recognized due to the increasing awareness of psychiatric complications in strokes and the utilization of psychotropic medications towards recovery [11–15, 23]. In our study, we found an association between greater functional deficit recovery and any psychotropic medication use (pre- or poststroke) in ischemic stroke compared to control, which leads to an important suggestion that psychotropic medication use may be beneficial in ischemic stroke. Our data also suggests that overall median mRS was still favorable (mRS score ≤ 2) for each group and that psychotropic medication use does not appear to increase the risks of MACE.

There are important implications related to prepsychotropic medication use in ischemic stroke. Preexisting SSRI use and poststroke recovery have not been widely studied. Thus far, there have been two studies, with one study looking at discharge mRS from the index admission [20] and one looking at the risk of stroke mortality and morbidity within 30 days [16]. In these studies, pre-SSRI use in ischemic stroke patients was associated with good clinical outcomes at early follow-up following acute ischemic stroke and that prestroke SSRI use was not associated with an increased risk of severe stroke or mortality within 1 month, respectively. Moreover, although the initiation of SSRI in an acute ischemic stroke and its effect on poststroke recovery has been studied in several randomized control studies, the results are conflicting [11, 13–15] as only one study with a smaller sample size was able to demonstrate a positive effect [11]. Future studies may benefit from evaluating prestroke measures, including prestroke psychotropic medication use as it may potentially complement other treatment strategies to improve stroke recovery [20]. Another important implication is the role of female gender in ischemic stroke and prepsychotropic medication use. In a pooled analysis of 19,652 patients, women were observed to have a higher disability and lesser quality of life following an ischemic stroke compared to men [24]. Furthermore, it is known that women have a higher prevalence of poststroke depression compared to men [25]. Poststroke depression is a serious and yet common complication of stroke, with more severe symptoms of poststroke depression occurring in women, which can be associated with higher mortality rates [23].

Poststroke recovery is known to involve activation of the mechanisms for plasticity in adjacent neurons through long-term potentiation (LTM) [26, 27]. Several neurotransmitters are known to be involved in LTM and the modulation of motor recovery after brain injury, including norepinephrine [6, 28, 29], dopamine [7, 8], and serotonin [9–11]. Further future prospective studies are needed to investigate the potential effects of preischemic stroke psychotropic medications targeting more than one neurotransmitter (such as SNRI targeting both serotonin and norepinephrine reuptake, for example) on the effects of long-term poststroke recovery.

The strengths of the study were independent reassessment of the clinical diagnoses by a trained neurologist, additional adjudication of uncertain cases by a double board-certified vascular neurologist and psychiatrist, and the case resolution by a general agreement by the group. An expectation bias was attempted to be minimized by classifying the study groups, blind to any follow-up data. Our study limitations are related to the retrospective study design, its relatively small sample size, and inclusion of the study population from a single tertiary care center, which may have attributed to bias. Another limitation includes lack of specific duration of the psychotropic medication use history in PPMU and PoMU. Our use of mRS as the outcome measures of stroke recovery can be potentially viewed as a limitation. However, the mRS is well known to be a reliable and valid measure of functional outcome [30–32] and has been used in a large, pragmatic clinical trial [13]. Other limitations include lack of patient reported outcome utilizations, including depression screen (e.g., PHQ-2), and lack of a stroke-specific, performance-based impairment index, such as the Fugl-Meyer assessment. However, these were impractical due to the retrospective study design.

## 5. Conclusion

PPMU in ischemic stroke is common; it can be beneficial in ischemic stroke in the long-term clinical outcome and is not associated with increased risks of MACE. Further studies are warranted to explore the effects of psychotropic medications in ischemic stroke.

## Figures and Tables

**(a):** 

Preischemic stroke psychotropic medication name	*n* = 69
Monotherapy	56
Sertraline	6
Amitriptyline	3
Venlafaxine	7
Duloxetine	0
Citalopram	11
Trazodone	9
Olanzapine	1
Nortriptyline	1
Fluoxetine	8
Pramipexole	1
Mirtazapine	2
Sinemet	1
Bupropion	1
Quetiapine	1
Paroxetine	4

Polytherapy	13
Postischemic stroke psychotropic medication use	*n* = 28
Monotherapy	25
Escitalopram	1
Quetiapine	1
Amitriptyline	1
Depakote	1
Citalopram	2
Trazodone	2
Paroxetine	1
Fluoxetine	16

Polytherapy	3

**(b):** 

Polytherapy	
Preischemic stroke case # (*n* = 13)	Medication names
1	Sertraline, methylphenidate
2	Duloxetine, quetiapine
3	Olanzapine, lithium
4	Citalopram, risperidone
5	Fluoxetine, trazodone
6	Divalproex sodium, trazodone, bupropion, methylphenidate
7	Amitriptyline, fluoxetine
8	Buspirone, sertraline
9	Quetiapine, mirtazapine
10	Citalopram, trazodone
11	Amitriptyline, mirtazapine, trazodone
12	Sertraline, trazodone
13	Escitalopram, trazodone
Postischemic stroke case # (*n* = 3)	Medication names
1	Citalopram, trazodone
2	Fluoxetine, trazodone
3	Olanzapine, trazodone

**Table 2:** Patient characteristics.

Characteristics	Prepsychotropic use (*n* = 69)	Non-PPMU (control +PoMU) (*n* = 254)	Unadjusted *P* value
Gender, female	47 (68.1%)	95 (37.4%)	<0.001
Age	66 (57-76)	67 (58-78)	0.443
Admission NIHSS	3 (1-8.5)	3 (1-7)	0.352
MRS on index presentation	0 (0-2)	0 (0-0)	<0.001
IV tPA on index presentation	12 (17.4%)	62 (24.4%)	0.26
Thrombectomy	9 (13.0%)	25 (9.8%)	0.442
Decompressive craniectomy	1 (1.4%)	7 (2.8%)	0.536
Length of stay	4 (3-7.5)	4 (3-7)	0.731
Stroke volume (ml)	1.4 (1.0-7.0)	1.6 (1.0-13)	0.312
Discharge destination			0.343
Home	32 (46.4%)	115 (45.3%)	
Acute rehab	26 (37.7%)	113 (44.5%)	
Skilled nursing facility	11 (15.9%)	26 (10.2%)	
Preexisting risk factors			
HTN	51 (73.9%)	177 (69.7%)	0.494
Dyslipidemia	47 (68.1%)	155 (61.0%)	0.280
DM	23 (33.3%)	75 (29.5%)	0.542
History of TIA/stroke	23 (33.3%)	66 (26.0%)	0.226
Atrial fibrillation	8 (11.6%)	49 (19.3%)	0.137
CAD	16 (23.2%)	54 (21.3%)	0.730
PVD	14 (20.3%)	46 (18.1%)	0.680
Preadmission medications			
Statins	42 (60.9%)	121 (47.6%)	0.051
Antihypertensives	47 (68.1%)	156 (61.4%)	0.307
Antidiabetics	17 (24.6%)	58 (22.8%)	0.753
Antiplatelet	31 (44.9%)	122 (48.0%)	0.647
Anticoagulation	5 (7.2%)	18 (7.1%)	0.964
Lesion side			0.756
Right	31 (44.9%)	12 (44.1%)	
Left	30 (43.5%)	103 (40.6%)	
Both	7 (10.1%)	29 (11.4%)	
Lesion circulation			0.001
Anterior	46 (66.7%)	143 (56.3%)	
Posterior	13 (18.8%)	95 (37.4%)	
Both	9 (13.0%)	7 (2.8%)	
Hemorrhagic conversion	6 (8.7%)	22 (8.7%)	0.993

Data are median (IQ range) and *n* (%).

**Table 3:** Outcome events within 365 days from the initial ischemic stroke.

Outcome events within 365 days				
	Control (*n* = 168)	Prepsychotropic use (*n* = 58)	Postpsychotropic use (*n* = 20)	*P* value
NIHSS	0 (0-2)	1 (0-3)	1.5 (0-6)	0.035
Delta NIHSS∗	1 (0-4)	3 (0-6)	5 (0-9)	0.086
Good clinical recovery	140 (82.8%)	48 (82.8%)	16 (80.0%)	0.891
mRS	1 (0-2)	2 (0-3)	2 (1-3)	0.013
MACE	18 (10.7%)	6 (10.3%)	1 (5.0%)	0.831
Myocardial infarction	2 (1.2%)	1 (1.7%)	0 (0.0%)	1.000
TIA or stroke	15 (8.9%)	5 (8.6%)	1 (5.0%)	1.000
Cardiovascular death	1 (0.6%)	0 (0.0%)	0 (0.0%)	1.000

Data are median (IQ range) and *n* (%); MACE: major adverse cardiovascular event. ∗Higher numbers indicate greater improvement from discharge.

## Data Availability

The data used to support the findings of this study are available from the corresponding author upon request to investigators who have received ethical clearance from their host institution review board.
